# Mechanistic insights into the role of calcium in the allosteric regulation of the calmodulin-regulated death-associated protein kinase

**DOI:** 10.3389/fmolb.2022.1104942

**Published:** 2022-12-19

**Authors:** Xiaolong Li, Bo Li, Jun Li, Mingyuan Yang, Yushu Bai, Kai Chen, Ziqiang Chen, Ningfang Mao

**Affiliations:** ^1^ Department of Orthopedics, Changhai Hospital, Naval Medical University, Shanghai, China; ^2^ Department of Orthopedics, Tongji Hospital, School of Medicine, Tongji University, Shanghai, China

**Keywords:** calcium, calmodulin, death-associated protein kinase, allostery, molecular dynamics simulation

## Abstract

Calcium (Ca^2+^) signaling plays an important role in the regulation of many cellular functions. Ca^2+^-binding protein calmodulin (CaM) serves as a primary effector of calcium function. Ca^2+^/CaM binds to the death-associated protein kinase 1 (DAPK1) to regulate intracellular signaling pathways. However, the mechanism underlying the influence of Ca^2+^ on the conformational dynamics of the DAPK1−CaM interactions is still unclear. Here, we performed large-scale molecular dynamics (MD) simulations of the DAPK1−CaM complex in the Ca^2+^-bound and-unbound states to reveal the importance of Ca^2+^. MD simulations revealed that removal of Ca^2+^ increased the anti-correlated inter-domain motions between DAPK1 and CaM, which weakened the DAPK1−CaM interactions. Binding free energy calculations validated the decreased DAPK1−CaM interactions in the Ca^2+^-unbound state. Structural analysis further revealed that Ca^2+^ removal caused the significant conformational changes at the DAPK1−CaM interface, especially the helices α1, α2, α4, α6, and α7 from the CaM and the basic loop and the phosphate-binding loop from the DAPK1. These results may be useful to understand the biological role of Ca^2+^ in physiological processes.

## 1 Introduction

Calcium (Ca^2+^) ions, as one of the most essential metals in human cells, are involved in multiple physiological process, including signal transduction, gene transcription, cell metabolism, cell growth and proliferation, and bioenergetics (Carafoli and Krebs, 2016; Martucci and Cancela, 2022; Yasuda et al., 2022). Due to the versatility and universality of Ca^2+^ signaling in the regulation of cellular function, its abnormality has been associated with many human diseases such as cardiovascular diseases, Alzheimer’s diseases, Parkinson’s diseases, diabetes, and cancer (Jomova et al., 2022; Qu et al., 2022; Shah et al., 2022).

The small and highly expressed Ca^2+^-binding protein calmodulin (CaM) acts as a primary effector of calcium function (Soderling and Stull, 2001). Upon Ca^2+^ binding, CaM is capable of interacting with hundreds of protein targets to regulate the wealth of intracellular signaling pathways. For example, CaM can bind to ∼15% of human protein kinases to modulate kinase cascades in response to calcium signaling (Simon et al., 2015; Tokumitsu and Sakagami, 2022), which is a possible link between Ca^2+^-dependent and phosphorylation-dependent signaling processes. In the Ras-PI3K signaling pathway, CaM forms a ternary complex consisting of K-Ras and phosphatidylinostide-3-kinase *a* (PI3Kα) to promote full PI3Kα activation by oncogenic K-Ras, highlighting the role of CaM in PI3K signaling (Nussinov et al., 2017; Ni et al., 2018; Zhang et al., 2018). CaM consists of two domains, the N- and C-terminal lobes (Soderling and Stull, 2001; Andrews et al., 2020), which is connected by a flexible linker. CaM presents two conformational architectures, including the collapsed and the extended forms, because of the flexibility of CaM’s linker.

The death-associated protein kinase 1 (DAPK1) is one of the binding targets for CaM (Cohen et al., 1997; Inbal et al., 2000). DAPK1, located in human chromosomal locus 9q34.1, is a member of the DAPK family that belongs to the serine/threonine kinase (STK) superfamily. The full-length sequence of DAPK1 has 1,430 residues (Farag and Roh, 2019), which consists of the catalytic domain (CD), the autoregulatory domain (ARD), eight ankyrin repeats, two P-loop motifs, the cytoskeletal binding domain, the death domain, and the serine-rich C-terminal tail. The CD controls the catalytic activity of DAPK1, the ARD plays an important role in the recognition of CaM, and the remaining domains are involved in localization. The ARD interacts with the CD, residing the kinase in its autoinhibited state. CaM binding to the ARD triggers large conformational arrangements of DAPK1 through the disruption of the CD−ARD association, generating a constitutively active kinase.

**FIGURE 1:**
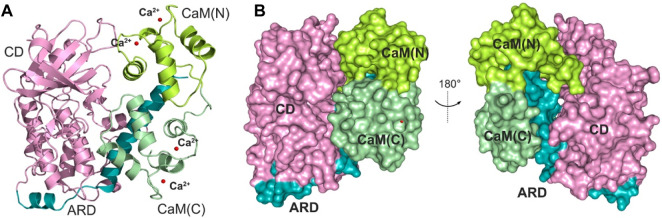
**(A)** Cartoon representation of the crystal structure of the DAPK1−CaM complex (PDB ID: 2X0G). The catalytic domain (CD) and the autoregulatory domain (ARD) of the DAPK1 are colored by pink and teal, respectively. The N- and C-terminal CaM are colored by limon and palegreen, respectively. Ca^2+^ ions are shown by red spheres. **(B)** Surface representation of the DAPK1−CaM structural complex.

The determination of an X-ray crystal structure of the CD and ARD portions of the DAPK1 in complex with the CaM provides the molecular basis of CaM-dependent regulation of DAPK1 (Figure 1A) (de Diego et al., 2010). CaM contains four Ca^2+^-binding sites, including two in the N-terminal domain, CaM(N) and two in the C-terminal domain, CaM(C). CaM can sense physiological Ca^2+^ concentrations and acts as an intracellular Ca^2+^ sensor (Soderling and Stull, 2001). In the Ca^2+^-bound state, both the CaM(N) and the CaM(C) adopts an extended conformation, exposing the hydrophobic cleft. The exposed hydrophobic cleft can engage with other proteins such as DAPK1. In the DAPK1−CaM complex, both the CD and the ARD of the kinase interact with the CaM (Figure 1B). Biochemical assays showed the decreased Ca^2+^ level resulted in a reduced catalytic efficiency of DAPK1 (de Diego et al., 2010). However, the mechanism underlying the effect of Ca^2+^ on the conformational dynamics of the DAPK1−CaM interaction remained unclear.

In the present study, the influence of the conformational dynamics of the DAPK1−CaM complex exerted by Ca^2+^ was explored using multiple, microsecond-length molecular dynamics (MD) simulations, which were carried out on the DAPK1−CaM complex in the presence and absence of Ca^2+^. A comprehensive analysis of the conformational changes of the DAPK1−CaM complex without Ca^2+^ allowed us to evaluate the effect of Ca^2+^ binding on the protein dynamics and to reveal the biological role of Ca^2+^ in physiological processes.

## 2 Materials and methods

### 2.1 Initial structural model

The X-ray crystal structure of DAPK1 that contains the CD and ARD domains in complex with Ca^2+^/CaM was obtained from the Protein Data Bank (PDB ID: 2X0G) (de Diego et al., 2010). The coordinates of the missing residues of CaM (residues 74–84) at the flexible linker were built using the MODELLER program (Fiser and Sali, 2003). All Ca^2+^ were removed from CaM to simulate the Ca^2+^-unbound DAPK1−CaM system. The protonation states for the ionizable residues at pH = 7 were used. For the histidine residues, the protonation states were determined based on the PROPKA calculation (Rostkowski et al., 2011). All the histidine residues in the DAPK1−CaM complex were set in a neutral state, however, the HID or HIE forms for the histidine residue was chosen based on the local hydrogen bonding network.

### 2.2 MD simulations

The *tleap* module of AMBER 18 was used to add the hydrogen atoms for the DAPK1−CaM complex (Salomon-Ferrer et al., 2013). The force field parameters for Ca^2+^ were downloaded from the AMBER parameters database (http://www.pharmacy.manchester.ac.uk/bryce/amber), using the value of van der Waals radius of R^*^ = 1.79 Å and the well depth of *ε* = 0.0140 kcal/mol. This force field parameter for the Ca^2+^ used in the MD simulations have been reported to reproduce the Ca^2+^−residue interactions in the X-ray crystal structure (Lawrenz et al., 2010).

Both systems were embedded in a truncated octahedron box of the TIP3P water molecules with a 10 Å buffer (Jorgensen et al., 1983). The Na^+^ counter-ions were added to maintain the electroneutrality of both systems, and then 0.15 mol/L NaCl was added to simulate physiological conditions. Energy minimizations and MD simulations were performed in an isothermal isobaric ensemble (*NPT*) with periodic boundary conditions using the *CUDA* module. Structure optimizations were carried out in a stepwise manner with the harmonic force restraint of the DAPK1−CaM complex and Ca^2+^ and the whole system then allowed to fully move. Energy optimizations were performed using the steepest descent method for the first 5,000 steps and then the conjugated gradient method for the subsequent 10,000 steps. Then, both systems were heated from 0 to 300 K in 100 ps. This was followed by constant temperature equilibration at 300 K for 500 ps at the canonical ensemble (*NVT*). In the production runs, 1,000 ns simulations were carried out, which were repeated three times using random velocities. The long-range electrostatic interactions were treated by the particle mesh Ewald method (Darden et al., 1993). A cut-off of 10 Å was used for short-range electrostatics and van der Waals interactions. All covalent bonds involving hydrogen atoms were constrained using the SHAKE method (Ryckaert et al., 1977). An integration step of 2 fs was used.

### 2.3 Cross-correlation (*C*_*ij*_) analysis

The cross-correlation matrix (*C*_*i*j_) between the fluctuations of the Cα atoms of the DAPK1–CaM was used to show the coupling of the motions between the protein residues (Li et al., 2020, Li et al., 2021; Shi et al., 2022). *C*_*ij*_ was calculated using following equation,

$$C\left(i,j\right)=\frac{c\left(i,j\right)}{c{\left(i,i\right)}^{1/2}c{\left(j,j\right)}^{1/2}}$$

Positive *C*_*ij*_ values mean the two atoms *i* and *j* moving in the same direction, while negative *C*_*ij*_ values describe anti-correlated motions between the two atoms *i* and *j.*

### 2.4 Community network analysis

The correlation *C*_*ij*_ data were further used to weight edges and compute the edge distance $D_{ij}$ using the following equation (Sethi et al., 2009; Saha et al., 2020; Zhuang et al., 2022a; 2022b), which represents the possibility of information flow.

$$D_{ij}=-\log\left(\left|C_{ij}\right|\right)$$

The community network was defined as a set of nodes (Cα atoms of the DAPK1–CaM complex), which was described by *CC*_*ij*_ weighted edges between two nodes within a cutoff distance of 4.5 Å for >75% of the simulated trajectory. The distribution of communities within the whole protein was decided and optimized using the Girvan-Newman algorithm. Communities harboring residues less than three were omitted.

### 2.5 Binding free energy calculations

The molecular mechanisms–generalized Born surface area energy calculations (MM–GBSA) were performed using the following equations (Wang et al., 2019; Liu et al., 2022a; Liu et al., 2022b; He et al., 2022; Zhu et al., 2022).

$$∆G_{binding}=∆G_{complex}-\left[∆G_{protein}+∆G_{ligand}\right]$$

$$∆G_{binding}=∆GE_{gas}+∆G_{solvation}-T∆S$$

$$∆E_{gas}=∆E_{vdW}+∆E_{ele}$$

$$∆G_{solvation}=∆G_{GB}+∆G_{nonpolar}$$

$$∆G_{nonpolar}=\gamma \times SΑSΑ+b$$

The Δ*E*_gas_, Δ*E*_vdW_, Δ*E*_ele_, Δ*G*_*s*olvation_, Δ*G*_GB_, and Δ*G*_nonpolar_ terms were gas energy, van der Waals energy, electrostatic energy, solvation free energy, the polar energy, and the non-polar energy, respectively. The Δ*G*_nonpolar_ was calculated using the function of the solvent accessible surface area (SASA) with the *γ* value of 0.0072 kcal/(mol Å^2^) and the b value of 0 kcal/mol. The *T*Δ*S* energy item was not calculated due to the extremely long durations of normal mode analysis for large systems.

## 3 Results and discussion

### 3.1 Dynamic behavior of the structural complex

To investigate the overall system stability and time-dependent conformational dynamics of the DAPK1−CaM complex in the presence and absence of Ca^2+^, a total of 6 μs-length MD simulations were carried out in the explicit water environment using the AMBER 18 program. Both systems, including Ca^2+^-bound and-unbound DAPK1–CaM complexes, were simulated in three independent times using random velocities to achieve reliable statistics. The overall stability of protein complex and the time-dependent protein-protein interactions were analyzed through extracting structural complexes from the production trajectories at different time intervals (Lu et al., 2019, 2021; Jang et al., 2020; Maloney et al., 2021; Ni et al., 2021). Then, conformational changes of the DAPK1–CaM complex in the Ca^2+^-bound and-unbound forms were compared. Root-mean-square deviation (RMSD), principal component analysis (PCA), dynamical cross-correlation matrix (DCCM), radial pair distribution function g(r), community network analysis, and MM–GBSA binding free energy calculations were calculated throughout 1,000 ns time scale simulations.

**FIGURE 2:**
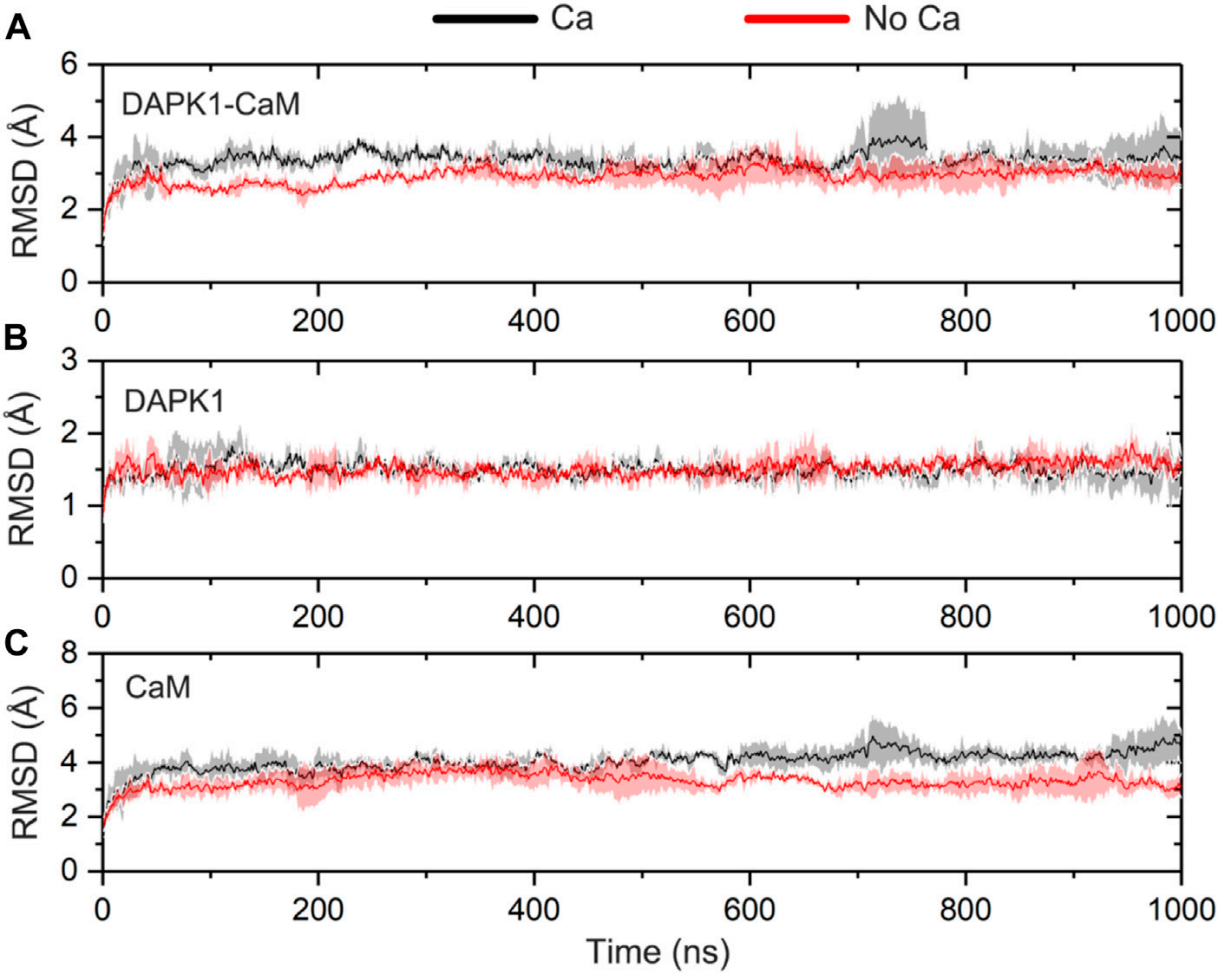
Time-dependent root-mean-square deviation (RMSD) of the Cα atoms for the DAPK1−CaM complex **(A)**, the independent DAPK1 **(B)** and CaM **(C)** in the three independent runs in the presence (black) and absence (red) of Ca^2+^. Transparent shades mean standard deviations.

To reveal the system convergence, the RMSD of the Cα atoms for the complex, DAPK1, and CaM was monitored with reference to the crystal structure (PDB ID: 2X0G) along the three independent simulations. In general, the complex (Figure 2A), DAPK1 (Figure 2B), and CaM (Figure 2C) in both systems were convergent in the early stage of simulations. Moreover, the RMSD values for the complex, DAPK1, and CaM were similar in both systems. For instance, in the Ca^2+^-bound state, the RMSD values for the complex, DAPK1, and CaM were 3.29 ± 0.39, 1.49 ± 0.18, and 4.04 ± 0.42 Å, respectively. In the Ca^2+^-unbound state, the RMSD values for the complex, DAPK1, and CaM were 3.02 ± 0.30, 1.51 ± 0.16, and 3.41 ± 0.41 Å, respectively. Together, these results suggested that the system stability of the DAPK1–CaM had no significant change in the absence of Ca^2+^ binding.

### 3.2 Principal component analysis (PCA)

To reveal the large-scale collective motions and the conformational interconversion of the DAPK1−CaM complex in the Ca^2+^-bound and-unbound states, PCA analysis of both simulated systems was performed (Masterson et al., 2011; An et al., 2021). The covariance matrix of the Cα atoms for the DAPK1−CaM complex was diagonalized to produce a set of eigenvalues and the corresponding eigenvectors. Each eigenvector is named the principal component (PC), which is related to an eigenvalue corresponding to the mean square fluctuation projected along the that eigenvector. The first several *P*Cs represent the overall fluctuations of the structural complex (Palermo et al., 2016). Each snapshot was first subjected to RMS-fit to the initial crystal structure of the DAPK1−CaM complex (PDB ID: 2X0G) as the same reference configuration. Then, all snapshots from MD simulations were projected into the collective coordinate space defined by the first two eigenvectors (PC1 and PC2), which reflected the essential conformational subspace sampled by the DAPK1−CaM complex in the Ca^2+^-bound and-unbound states.

**FIGURE 3:**
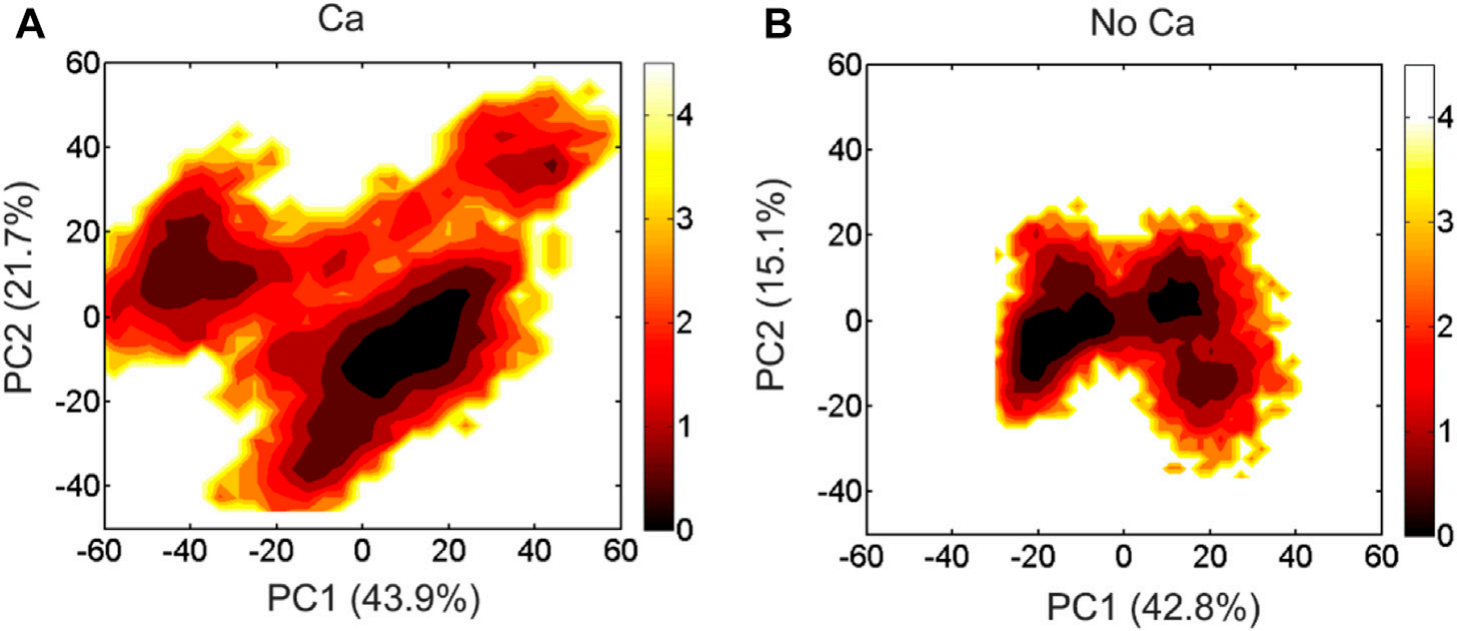
The free energy landscape of the PC1 and PC2 for the DAPK1−CaM complex in the Ca^2+^-bound **(A)** and-unbound **(B)** states. The unit of free-energy values is kcal/mol.

We performed PCA analysis of the DAPK1−CaM complex in the presence and absence of Ca^2+^ binding and found that the PC1 and PC2 represented ∼60% of variance in coordinates along the MD simulations in both systems. The free energy landscapes (FELs) of the PC1 and PC2 showed the distinct conformational space sampled by the DAPK1−CaM complex in the Ca^2+^-bound and-unbound states. As shown in Figure 3A, in the presence of Ca^2+^, the PC1 and PC2 plots sampled a broad distribution in the FELs, with the PC1 and PC2 values in the range of ∼ − 60 to ∼60 and ∼ − 40 to ∼60, respectively. However, in the absence of Ca^2+^ (Figure 3B), the PC1 and PC2 plots sampled a confined distribution, with the PC1 and PC2 values in the range of ∼ − 30 to ∼40 and ∼ − 30 to ∼20, respectively. Moreover, two major conformational substates of the DAPK1−CaM complex were observed in both the Ca^2+^-bound and-unbound states. Collectively, these results suggested that Ca^2+^ binding increased the dynamics and the conformational space of the DAPK1−CaM complex.

### 3.3 Coupled motions of protein intra-and inter-domains

**FIGURE 4:**
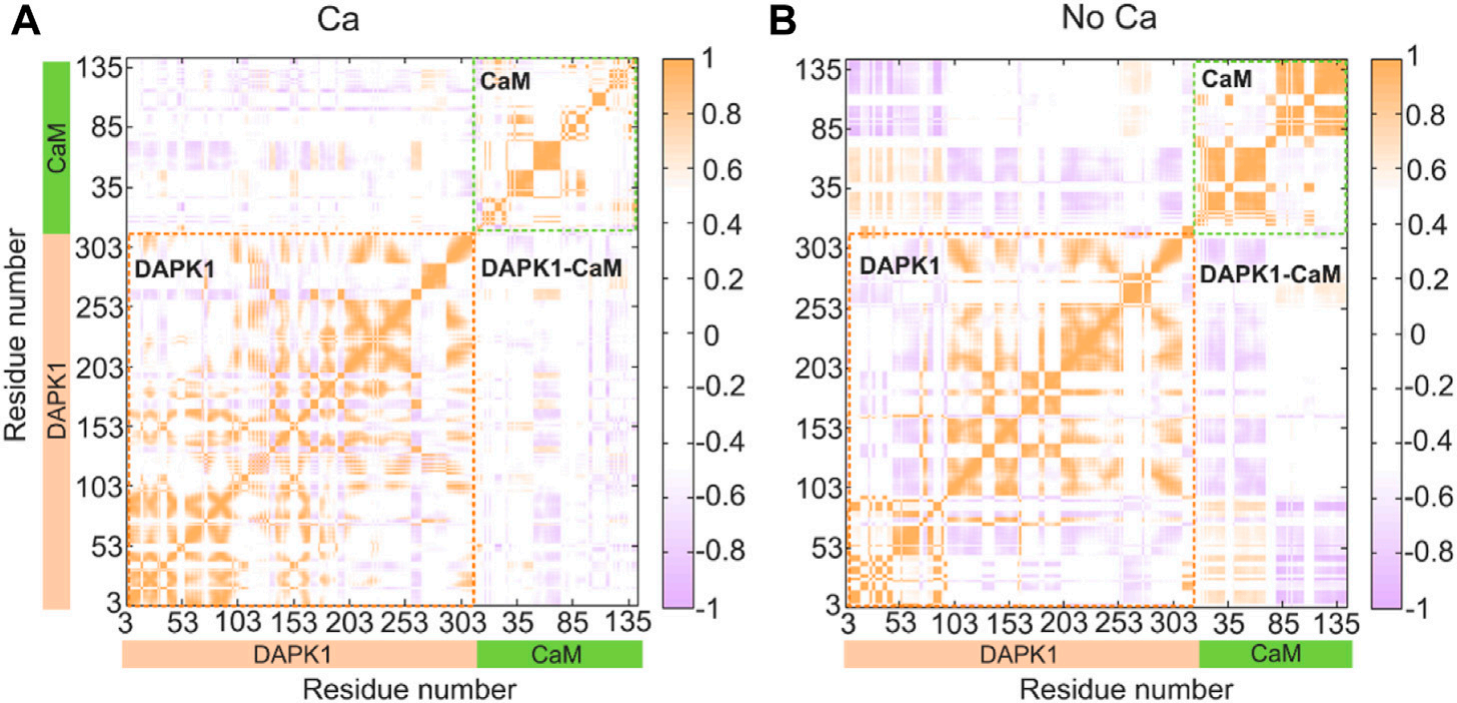
Intra- and inter-domain correlation of the DAPK1−CaM in the Ca^2+^-bound **(A)** and-unbound **(B)** states. Positive regions (orange) stand for correlated motions, while negative regions (light blue) represent anti-correlated motions. The absolute values of the coefficients (<0.4) are shown in white for clarity.

We next sought to explore how Ca^2+^ induces conformational changes of the DAPK1−CaM complex. The global intra- and inter-domain motions in the protein-protein complex involve the collective motions of the protein backbone atoms. To reveal the correlated or anti-correlated motions between different residues and domains within the DAPK1−CaM complex, the dynamical cross-correlation matrix (DCCM) for the Cα atoms of the structural complex were constructed and analyzed in both the Ca^2+^-bound and-unbound states. As shown in Figure 4, the DCCM plots showed the correlated or anti-correlated motions between residues within the protein complex, with the regions in orange representing the correlated motions and the regions in purple representing the anti-correlated motions and the density of the color corresponding to the intensity of correlated/anti-correlated motions.

In the presence of Ca^2+^ (Figure 4A), the intra-domain motions in both the independent DAPK1 and CaM showed mainly correlated motions, while the inter-domain motions between the DAPK1 and CaM underwent weak anti-correlated motions. However, in the absence of Ca^2+^ (Figure 4B), the intra-domain motions in the independent DAPK1 showed mixed correlated and anti-correlated motions. Although the intra-domain motions in the independent DAPK1 showed correlated motions without Ca^2+^ binding, the intensity of the correlated motions was stronger in the Ca^2+^-unbound state than in the Ca^2+^-bound state. Remarkably, compared to the inter-domain motions between the DAPK1 and CaM in the Ca^2+^-bound state, they underwent enhanced anti-correlation motions in the Ca^2+^-unbound state. The increase of the anti-correlation motions between the DAPK1 and CaM in the Ca^2+^-unbound state indicated that the Ca^2+^-unbound CaM might disassociate from the DAPK1, which can be assessed by the following binding free energy calculations.

### 3.4 Community network analysis

**FIGURE 5:**
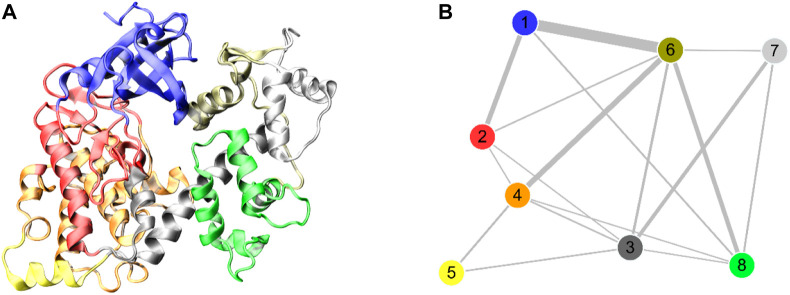
The three-dimensional **(A)** and two-dimensional **(B)** visualizations of the difference contact networks for the DAPK1−CaM complex between the Ca^2+^-bound and-unbound states. The communities are represented by circles with different colors and the widths of sticks connecting communities highlighting the intercommunity connection difference between the two systems.

To explore the effect of Ca^2+^ removal on the allosteric network of the DAPK1–CaM complex, the difference contact network analysis (dCNA) was performed (Li et al., 2022). The connected residues are considered as one community, which can be served as a synergistic functional unit of the protein. The dCNA analysis can unravel the intercommunity changes between difference conformational ensembles. The same communities are represented by spheres that are connected by sticks, whose width is corresponding to the intensity of interaction between different communities. Figure 5 shows the three-dimensional and two-dimensional visualizations of the dCNA between the Ca^2+^-bound and-bound states, respectively. We mainly focused on the connectivity between the interface of the DAPK1–CaM complex. The community one mainly contained the B-loop and the P-loop of the DAPK1, the community 3 was composed of the C-terminal ARD of the DAPK1, the community six included the helices α1 and α4 of the CaM(N), and the community 8 possessed the CaM(C) that contained the helices α5–α8. After the removal of Ca^2+^, the connectivity between the community 1 of the DAPK1 and the community six of the CaM(N), between the community 3 of the DAPK1 and the communities 6/7 of the CaM(N), and the community 4 of the DAPK1 and the community 7 of the CaM(N) decreased. These results suggested that the allosteric communication between the DAPK1 and CaM was reduced in the Ca^2+^-bound state, which would weaken the DAPK1–CaM interaction.

### 3.5 Binding free energy calculations

To further show the energetics of the DAPK1–CaM interactions in the Ca^2+^-bound and-unbound states, the binding free energy (ΔG_binding_) calculations were performed using the MM–GBSA method, which has been proved successfully to evaluate protein-ligand or protein-protein interactions (Liu et al., 2018; Wang et al., 2019, 2021, 2022). A total of 200 snapshots for the last 200 ns snapshots were selected from the MM–GBSA binding free energy calculations. As shown in Table 1, the ΔG_binding_ values in the Ca^2+^-bound and-unbound states were −112.98 ± 20.63 and −86.40 ± 18.79 kcal/mol, respectively. Thus, the binding free energy between DAPK1 and CaM was larger by −26.58 kcal/mol in the Ca^2+^-bound state than that in the Ca^2+^-unbound state, which suggested that the removal of Ca2+ would disfavor the DAPK1–CaM interactions.

**TABLE 1:** Binding free energy (kcal/mol) between DAPK1 and CaM in the Ca^2+^-bound and-unbound states.

	Ca^2+^-bound	Ca^2+^-unbound
Δ*E*_ele_	−1,650.34 ± 131.87	−1840.20 ± 141.32
Δ*E*_vdW_	2212190.77 ± 12.27	−191.96 ± 14.10
ΔG_SA_	−29.32 ± 1.52	−30.76 ± 2.02
ΔG_GB_	1728.12 ± 124.22	1945.76 ± 134.08
Δ*G*_binding_	−112.98 ± 20.63	−86.40 ± 18.79

### 3.6 Ca^2+^ coordination modes

**FIGURE 6:**
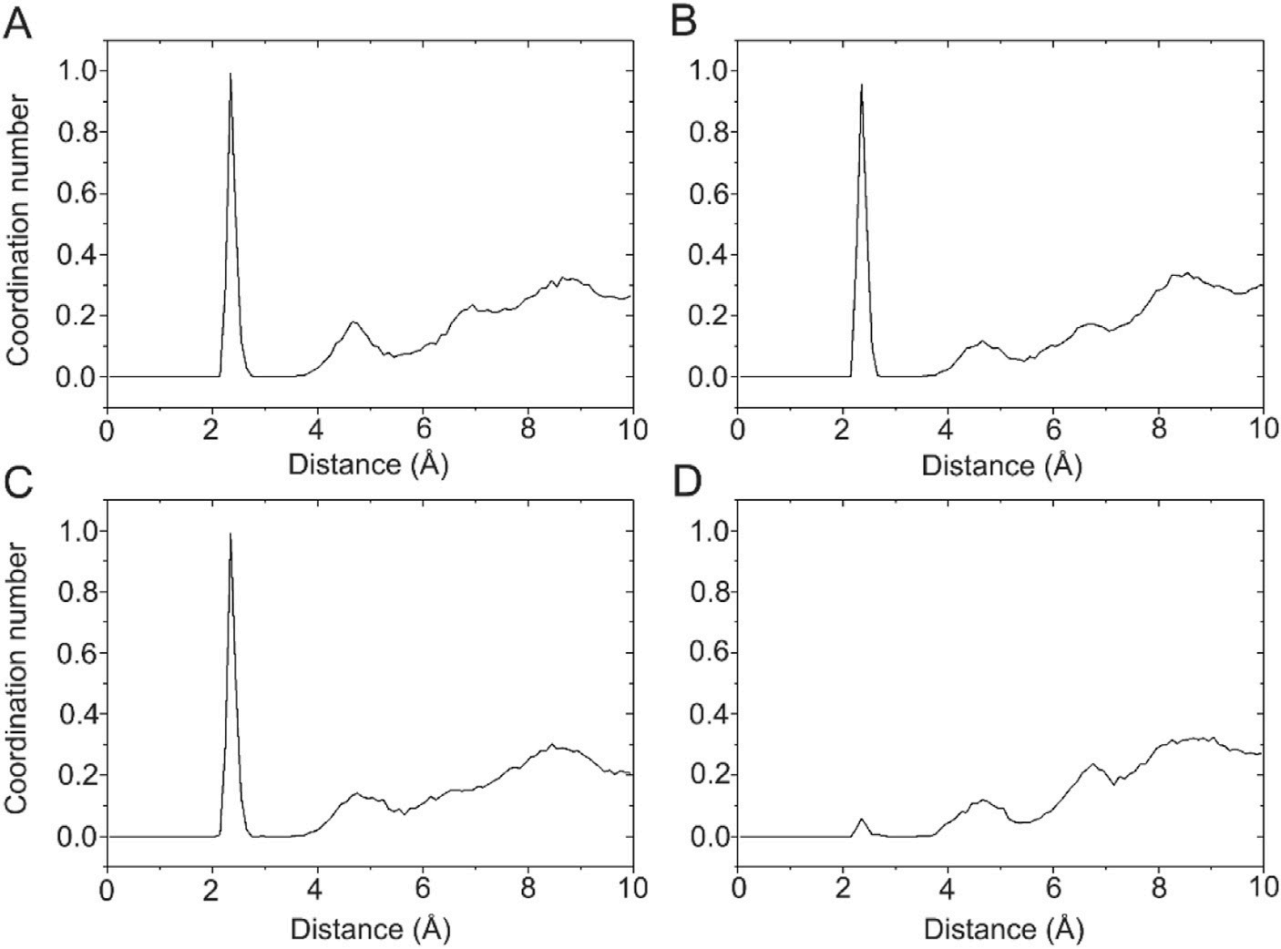
The coordination numbers of water molecules for the Ca^2+^ in the CS1 **(A)**, CS2 **(B)**, NS1 **(C)**, and NS2 **(D)** binding sites in the Ca^2+^-bound state.

Ca^2+^ has a radius of 0.99 Å, showing coordination flexibility with coordination numbers (CNs) ranging from six to eight and with an average CN of 7.3 (Kirberger et al., 2008). In the X-ray crystal structure of the DAPK1−CaM complex (PDB ID: 2X0G) (de Diego et al., 2010), there are four Ca^2+^ binding sites, including two in the C-terminus (CS1 and CS2) and two in the N-terminus (NS1 and NS2). However, during crystallization, crystal waters are often omitted. With this idea in mind, we calculated the radial pair distribution function g(r) between each Ca^2+^ and the water molecules. Radial pair distribution function g(r) can provide the probability of finding particles at a certain distance (Lu et al., 2013; Sameera et al., 2020). Figure 6 shows the radial pair distribution function g(r) derived from the MD simulations in the Ca^2+^-bound DAPK1−CaM complex. For the Ca^2+^ binding sites at the CS1, CS2, and NS1, all plots showed a sharp peak at 2.35 Å, which suggested that there was a high probability that the water molecules were coordinated to these Ca^2+^ during MD simulations. However, in the NS2 binding site, no sharp peak at 2.35 Å was observed, indicating that no water molecules were coordinated to Ca^2+^ in the NS2 binding site. Furthermore, the CNs of the water molecules for Ca^2+^ at the CS1, CS2, and NS1 binding sites were computed. As shown in Figure 6, one water molecule was coordinated to each Ca^2+^ at the CS1, CS2, and NS1 binding sites.

**FIGURE 7:**
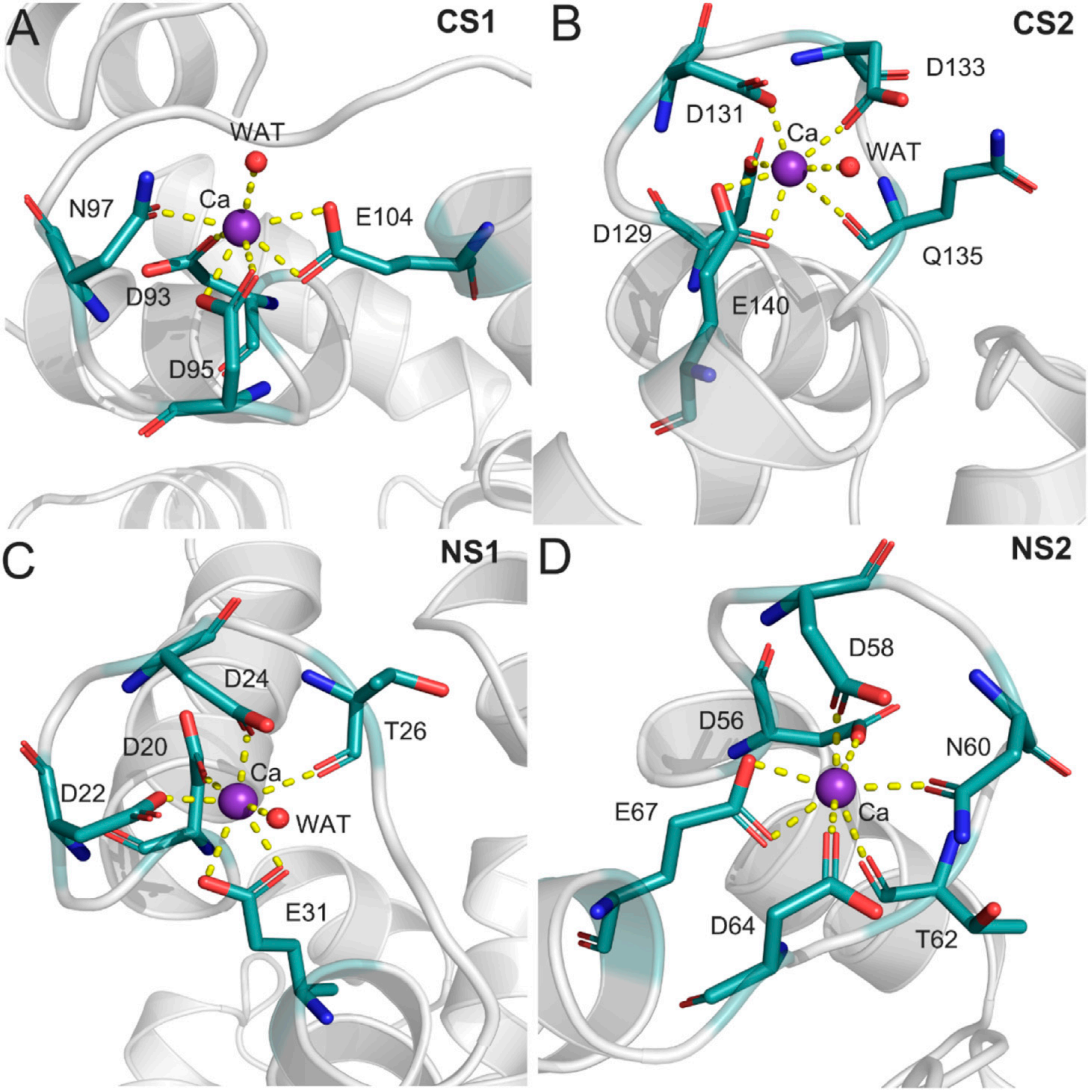
The detailed coordination interactions for the Ca^2+^ in the CS1 **(A)**, CS2 **(B)**, NS1 **(C)**, and NS2 **(D)** binding sites in the Ca^2+^-bound state.

To further characterize the Ca^2+^ coordination mode in each binding site, a detailed analysis of the MD trajectories was performed using cluster analysis. Figure 7 shows the most representative structure obtained from the MD trajectories and the detailed coordination mode for each Ca^2+^ binding site. Each Ca^2+^ has a CN of 7 from the CaM. In the CS1 binding site (Figure 7A), Ca^2+^ was coordinated by one oxygen atom from the sidechain Asn97, one oxygen atom from the sidechain Asp93, two oxygen atoms from the sidechain Asp95, two oxygen atoms from the sidechain Glu104, and one water oxygen atom. In the CS2 binding site (Figure 7B), Ca^2+^ was coordinated by one oxygen atom from the sidechain Asp129, one oxygen atom from the sidechain Asp131, one oxygen atom from the sidechain Asp133, one oxygen atom from the sidechain Gln135, two oxygen atoms from the sidechain Glu140, and one water oxygen atom. In the NS1 binding site (Figure 7C), Ca^2+^ was coordinated by one oxygen atom from the sidechain Asp20, one oxygen atom from the sidechain Asp22, one oxygen atom from the sidechain Asp24, one oxygen atom from the backbone Thr26, two oxygen atoms from the sidechain Glu31, and one water oxygen atom. In the NS2 binding site (Figure 7D), Ca^2+^ was coordinated by one oxygen atom from the sidechain Asp56, one oxygen atom from the sidechain Asp58, one oxygen atom from the sidechain Asn60, one oxygen atom from the backbone Thr62, one oxygen atom from the sidechain Asp64, and two oxygen atoms from the sidechain Glu67. These exquisite coordinated networks between Ca^2+^ and CaM render the CaM in a restrained conformational change when it binds to the DAPK1 protein. Thus, CaM would undergo large conformational changes after the removal of Ca^2+^ from each binding site, which was unfavorable for the binding to DAPK1 as revealed by the MM−GBSA binding free energy calculations.

### 3.7 Conformational changes due to Ca^2+^ removal from CaM

**FIGURE 8:**
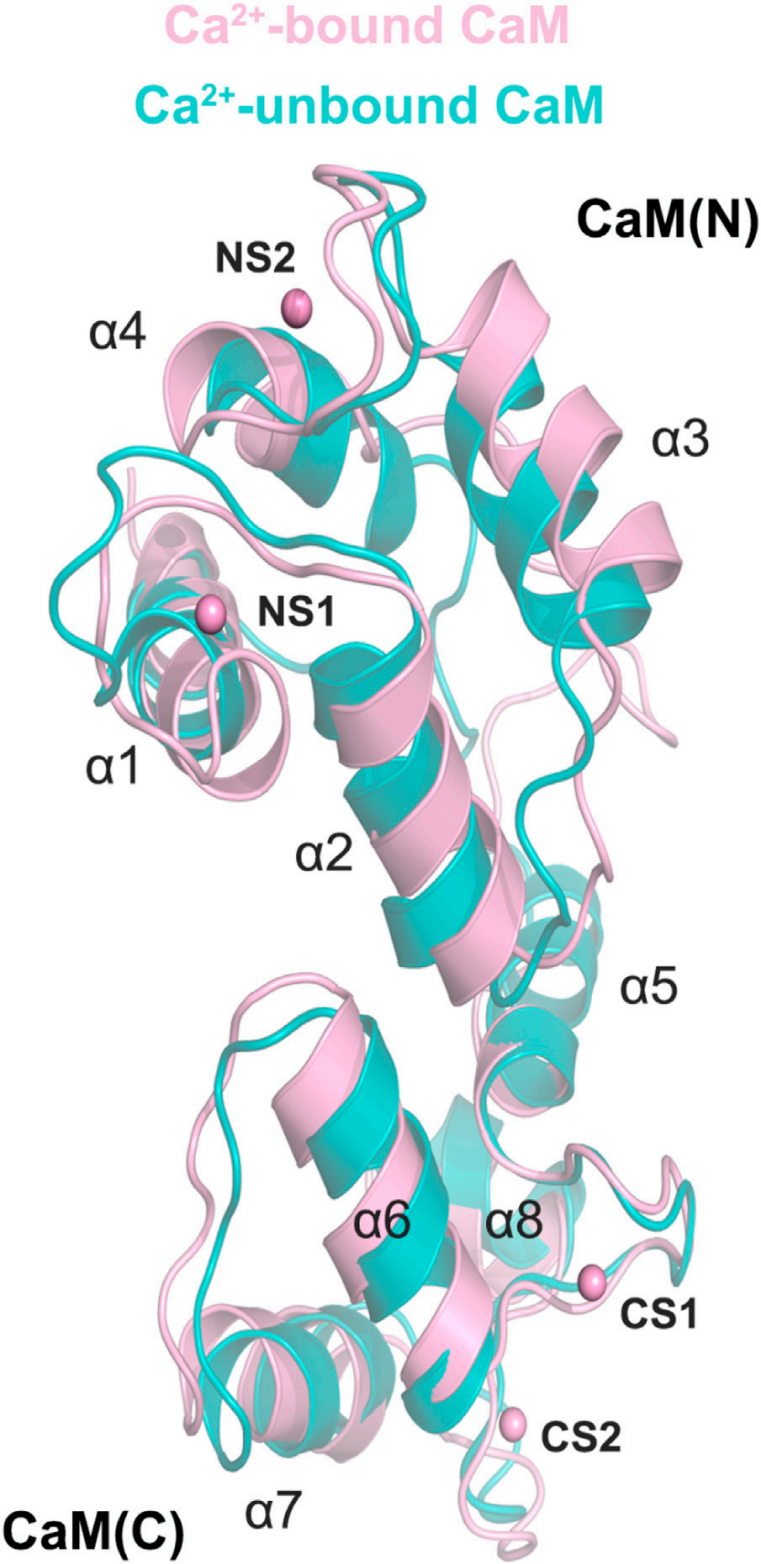
Structural overlapping of the CaM in the Ca^2+^-bound and-unbound states.

To investigate how Ca^2+^ removal affected the detailed conformational changes of the CaM and the DAPK1−CaM complex, the most representative structural complexes in both the Ca^2+^-bound and-unbound states were extracted using cluster analysis of MD trajectories (Shao et al., 2007). We first overlapped the independent CaM from MD simulations in both systems. The RMSD of the CaM between the Ca^2+^-bound and-unbound states was 2.07 Å. As shown in Figure 8, compared to the Ca^2+^-bound state, the appreciable conformational rearrangements of the CaM were found in the helices α1, α3, α4, α7 and the loop that connects the helices α1 to α2. According to the X-ray crystal structure of the DAPK1−CaM complex (PDB ID: 2X0G), the helices α1, α4, α7 and the loop that connects the helices α1 to α2 from the CaM are involved in the interactions with both the CD and the ARD of DAPK1. Thus, the disturbed conformational changes at the CaM interface would allosterically affect the association with the DAPK1. As a result, Ca^2+^ may serves an allosteric modulator of DAPK1−CaM protein-protein interaction (PPI), which has been an area of intensive research for PPI drug discovery (Ni et al., 2019, 2022).

**FIGURE 9:**
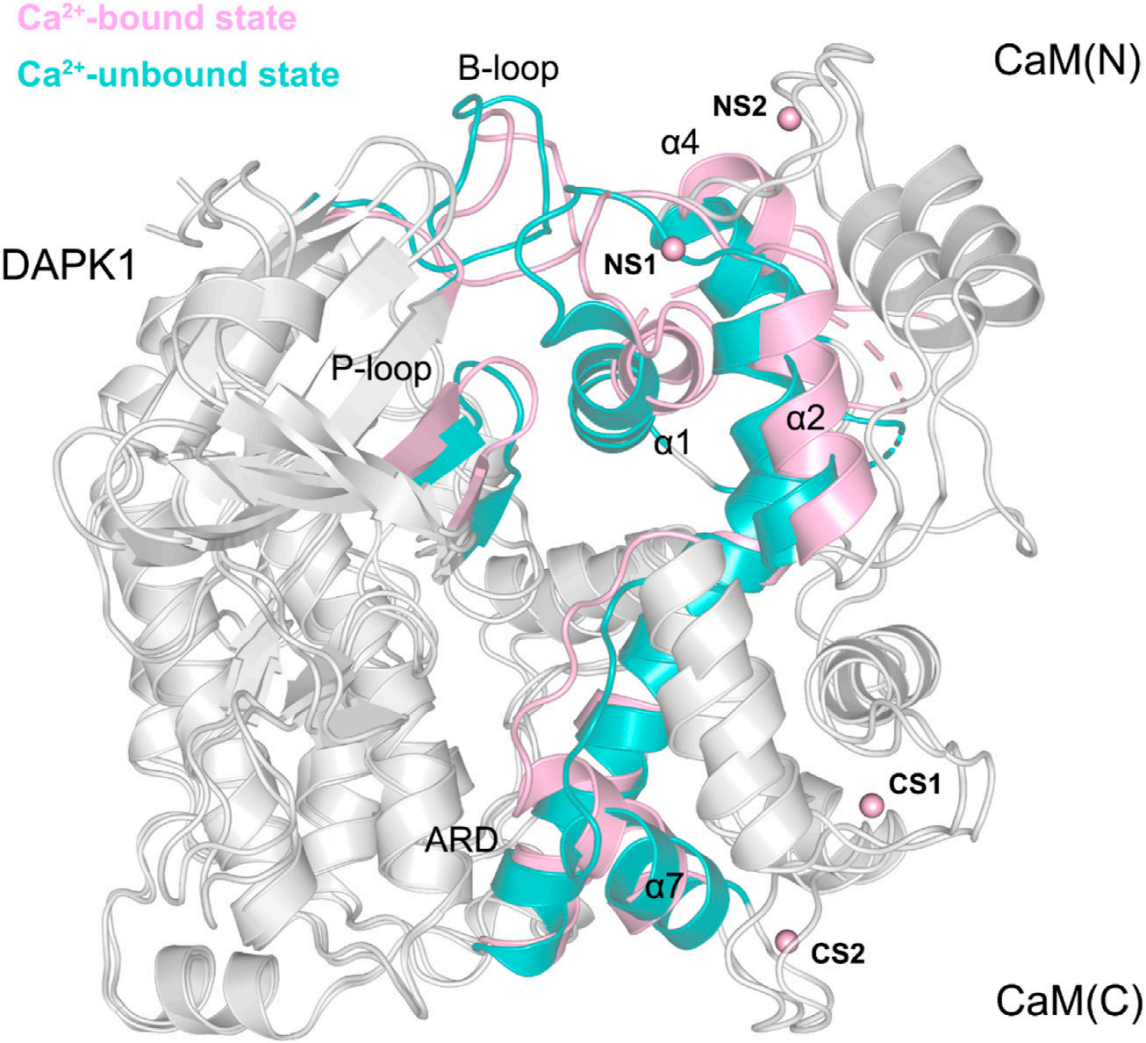
Structural overlapping of the DAPK1−CaM complex in the Ca^2+^-bound and-unbound states.

To further reveal the effect of Ca^2+^ removal on the conformational dynamics of the DAPK1−CaM complex, we superimposed the structural complexes in both the Ca^2+^-bound and-unbound states. As shown in Figure 9, compared to the DAPK1−CaM in the Ca^2+^-bound state, the removal of Ca^2+^ allosterically changed the conformations of the DAPK1 at the basic loop (B-loop) and the phosphate-binding loop (P-loop), while the C-terminal ARD of the DAPK1 underwent little conformational changes. Indeed, the B-loop and the P-loop of the DAPK1 are involved in the interactions with the helices α1 and α4 and the loop connecting the helices α1 to α2 of the CaM. The C-terminal ARD of the DAPK1 engages with the helices α2 and α7. Therefore, the disruption of the interface of the DAPK1-CaM complex after Ca^2+^ removal would disassociate the binding of Ca^2+^-free CaM to the DAPK1, which has been revealed by the above binding free energy calculations. As a result, the catalytic efficiency of DAPK1 would reduce without the regulation of CaM.

## 4 Conclusion

The present MD simulations have offered a mechanistic insight into the regulation of the DAPK1−CaM interaction by Ca^2+^ at the atomic level. Based on the simulation results, we showed that each of the four Ca^2+^ at the CaM forms a sevenfold coordination paradigm with amino acids residues or water molecules. The removal of Ca^2+^ had a minor effect on the overall conformational dynamics of the DAPK1−CaM complex, but the anti-correlated inter-domain motions between DAPK1 and CaM increased in the Ca^2+^-unbound state. The MM−GBSA binding free energy calculations showed that the ΔG_binding_ between the DAPK1 and CaM was unfavorable in the Ca^2+^-unbound state, indicating that the Ca^2+^-free CaM can dissociate from the DAPK1. Further structural investigation revealed that the conformations of the B-loop and the P-loop at the DAPK1 were allosterically disturbed in the Ca^2+^-unbound state. For the CaM, the conformational changes were observed at the helices α1, α4, α7 and the loop that connects the helices α1 to α2 after the removal of Ca^2+^. Thus, the interface of the DAPK1−CaM interaction was disrupted in the Ca^2+^-unbound state, which would unbind the Ca^2+^-free CaM to the DAPK1. The resulting unbinding of the CaM to the DAPK1 would reduce the catalytic efficiency of the DAPK1. These results were beneficial to understand the role of Ca^2+^ in the regulation of CaM-dependent modulation of DAPK1 catalytic activity.

## Data Availability

The original contributions presented in the study are included in the article/supplementary material, further inquiries can be directed to the corresponding authors.
